# Objective validation of the Crohn's Anal Fistula Quality of Life questionnaire using quantitative MRI fistula volume in perianal fistulising Crohn's disease

**DOI:** 10.1111/codi.70653

**Published:** 2026-10-17

**Authors:** Easan Anand, Theo Pelly, Uday Patel, Rory Callan, Reshma Kanani, Ailsa Hart, Phil Tozer, Phillip Lung

**Affiliations:** ^1^ St Mark's, the National Bowel Hospital, Central Middlesex London UK; ^2^ Department of Surgery & Cancer Imperial College London London UK; ^3^ Department of Metabolism, Digestion & Reproduction Imperial College London London UK

**Keywords:** biomarker, CAF‐QoL, fistula, MRI, perianal Crohn's, quality of life, validation, volume

## Abstract

**Background:**

The Crohn's Anal Fistula Quality of Life (CAF‐QoL) questionnaire is the first disease‐specific patient‐reported outcome measure (PROM) for perianal fistulising Crohn's disease (pfCD). However, validation against objective measures of disease burden is limited. We evaluated the relationship between CAF‐QoL and quantitative MRI fistula volume to determine whether MRI‐derived disease burden supports the construct validity of this PROM.

**Methods:**

Adults with pfCD were prospectively recruited from a tertiary referral centre. Patients underwent pelvic MRI and completed CAF‐QoL within 3 months of imaging. MRI fistula volume was independently quantified by two radiologists using ITK‐SNAP, with concurrent scoring of the Van Assche Index (VAI). Clinical disease activity was assessed using the Perianal Disease Activity Index (PDAI). Associations between MRI measures, CAF‐QoL and PDAI were evaluated cross‐sectionally and longitudinally.

**Results:**

Thirty‐eight patients were included. MRI fistula volume correlated with both CAF‐QoL (*ρ* = 0.40, *p* = 0.03) and PDAI (*ρ* = 0.59, *p* < 0.001). Patients achieving CAF‐QoL remission had significantly smaller fistula volumes than those with active disease (279 vs. 965 mm^3^, *p* = 0.04). Longitudinal changes in fistula volume correlated with changes in CAF‐QoL (*ρ* = 0.61, *p* = 0.02) and PDAI (*ρ* = 0.74, *p* ≤ 0.01). There was no significant association between the VAI and either CAF‐QoL or PDAI. Inter‐observer agreement for MRI volume measurement was excellent (ICC = 0.995).

**Conclusion:**

Quantitative MRI fistula volume provides objective construct validation of the CAF‐QoL questionnaire and captures clinically meaningful disease burden in pfCD. Combined assessment of MRI fistula volume and CAF‐QoL may provide complementary imaging and patient‐reported endpoints for clinical practice and future therapeutic trials. Larger prospective multicentre studies are warranted.

**What does this paper add to the literature?**

Quantitative MRI fistula volume provides the first objective validation of the Crohn's Anal Fistula Quality of Life questionnaire in perianal fistulising Crohn's disease. Volumetric assessment correlated with both clinical activity and patient‐reported quality of life, supporting its potential use as a reproducible imaging biomarker and a complementary endpoint for future clinical trials.

## INTRODUCTION

Perianal fistulising Crohn's disease (pfCD) is one of the most debilitating manifestations of Crohn's disease, profoundly affecting pain, sexual function, body image and social participation [1, 2]. Persistent drainage, recurrent sepsis, and repeated interventions result in substantial impairment in health‐related quality of life (QoL) [2]. Historically, QoL in pfCD has been inferred using clinician‐derived, non‐validated instruments such as the Perianal Disease Activity Index (PDAI) [3] and the Fistula Drainage Assessment (FDA) [4] which do not fully capture the lived experience of patients. In contrast, Crohn's Anal Fistula‐Quality of Life (CAF‐QoL) scale is a Patient‐Reported Outcome Measure (PROM) that was specifically developed with patients and clinicians to reflect domains most relevant to pfCD [5]. Subsequent psychometric validation has established a cut‐off score of less than or equal to 37 to indicate quality of life ‘remission’ and refinement into a concise 9‐item short‐form (SF) version [6]. Consensus studies have recommended inclusion of CAF‐QoL with clinical and radiological endpoints in future clinical trials and research studies [7, 8, 9].

MRI is the reference standard for assessing complex fistula anatomy and monitoring treatment response in pfCD [10]. Although semiquantitative MRI indices such as the Van Assche Index (VAI) and the newer, contrast‐dependent MAGnetic Resonance Novel Index for Fistula Imaging in Crohn's Disease (MAGNIFI‐CD) have standardised radiological assessment in research, their clinical use is limited by scoring complexity, ceiling effects, weak associations with patient‐reported outcomes [6] and limited sensitivity to fluctuations in disease activity [11, 12]. Quantitative radiology using MRI‐derived fistula volume offers a promising alternative by providing a rapid, reproducible, continuous measure of inflammatory burden that correlates with disease severity, without many of the limitations of MRI indices [11, 13, 14]. However, despite MRI's central role in the assessment of pfCD, quantitative imaging biomarkers have not previously been systematically evaluated against validated, disease‐specific patient‐reported outcome measures, limiting our understanding of how objective disease burden relates to the patient experience.

The Global Perianal Crohn's Disease Priority Setting Partnership has identified improved imaging assessment and better quality of life measurement as two of the top 10 priorities in research [15]. Despite these parallel developments, the relationship between objective MRI‐derived disease burden and disease‐specific patient‐reported quality of life remains poorly understood. We hypothesise that MRI‐derived fistula volume is a dynamic radiological biomarker of disease severity in pfCD that reflects patient‐reported quality of life. We therefore evaluated the construct validity of the CAF‐QoL questionnaire by examining its association with quantitative MRI fistula volume and the VAI. We also evaluated the reproducibility of MRI fistula volume as an objective imaging biomarker for routine assessment of pfCD.

## MATERIALS AND METHODS

### Study design and setting

This was a prospective observational cohort study conducted at a tertiary referral centre specialising in complex bowel pathology. Participants were recruited over an 18‐month period from October 2023 to April 2025. Patients were identified from a prospectively maintained perianal fistula outcomes database. No formal sample size calculation was undertaken because this was an exploratory prospective observational cohort. The study was reported in accordance with STROBE guidelines for observational studies [16].

### Participants

Adult patients (≥18 years) with pfCD were eligible for inclusion. Inclusion criteria were: (i) confirmed diagnosis of pfCD; and (ii) availability of at least one pelvic MRI scan performed within 3 months of completion of CAF‐QoL. Exclusion criteria were: (i) non‐fistulising perianal Crohn's disease; (ii) pouch‐related fistulae; and (iii) Class 4 perianal disease (post‐proctectomy status).

### Data collection

Prospectively collected clinical, radiological and patient‐reported variables were analysed.

### Baseline characteristics

Collected variables included demographics, smoking status, disease duration and Montreal classification. Medication exposure (immunosuppressants, biologics, corticosteroids) and prior perianal surgical interventions (seton placement, advancement flap, ligation of intersphincteric fistula tract [LIFT], fistulotomy) were recorded.

### Patient‐reported outcomes

Health‐related QoL was assessed using the CAF‐QoL questionnaire (total score 0–108, with higher scores indicating worse QoL) [5]. CAF‐QoL Domain scores, the 9‐item Short Form CAF‐QoL (SF‐CAF‐QoL) [6] and generic health status measures (EQ‐5D‐5L Level Sum Score [LSS] and Visual Analogue Scale [VAS] [17]) were also collected. Details of each patient‐reported outcome measure and scoring system are provided in Table 1.

**TABLE 1:** Patient‐reported outcome measures and scoring systems used in the study.

Instrument	Domain	Description	Score range	Interpretation
CAF‐QoL	A. Symptoms from fistula	Assesses symptom burden attributable to the fistula.	0–20	Higher scores indicate more frequent or severe symptoms (0 = no symptoms; 20 = symptoms several times a day)
	B. Impact of fistula treatment	Assesses the impact of fistula treatment on the patient.	0–12	Higher scores indicate a greater impact of treatment (0 = no effect; 12 = severe effect)
	C. Impact on quality of life	Assesses the impact of the fistula on quality of life during the preceding 6–8 weeks.	0–76	Higher scores indicate a greater negative impact on quality of life (0 = no impact; 76 = maximum impact)
	Total score	Sum of domains A–C.	0–108	Higher scores indicate worse disease‐specific quality of life
SF‐CAF‐QoL	—	Nine‐item abbreviated version assessing the physical, emotional and functional impacts of pfCD	0–36	Higher scores indicate worse disease‐specific quality of life
EQ‐5D‐5L	Level Sum Score (LSS)	Standardised measure of health status across five domains (mobility, self‐care, usual activities, pain/discomfort and anxiety/depression). The LSS is calculated by summing the five domain scores (each scored from 1 to 5)	5–25	Higher scores indicate poorer overall health status
EQ‐5D‐5L	Visual Analogue Scale (VAS)	Patient‐rated global assessment of overall health status using a visual analogue scale	0–100	Higher scores indicate better perceived health (0 = worst imaginable health; 100 = best imaginable health)

*Note*: For all CAF‐QoL measures and the EQ‐5D‐5L LSS, higher scores indicate poorer health status or quality of life. For the EQ‐5D‐5L VAS, higher scores indicate better perceived overall health.

Abbreviations: CAF‐QoL, Crohn's Anal Fistula Quality of Life questionnaire; EQ‐5D‐5L, EuroQol five‐dimension, five‐level questionnaire; LSS, Level Sum Score; SF‐CAF‐QoL, Short Form Crohn's Anal Fistula Quality of Life questionnaire; VAS, Visual Analogue Scale.

### Clinical and disease activity measures

Clinical disease activity was assessed using the PDAI. PfCD phenotype was classified according to the TOpClass system [18, 19].

### MRI acquisition

The MRI protocol included sagittal T2‐weighted and T2 fat‐saturated sequences, as well as coronal‐oblique and axial‐oblique T2 fat‐saturated sequences. Coronal and axial planes were angulated parallel and perpendicular to the anal canal. DICOM (Digital imaging and communications in medicine) images of the sequences were anonymised and retrieved from PACS. Only T2 fat‐saturated axial sequences were required for segmentation, although access to the remaining sequences was available if required.

### MRI evaluation and reliability

Perianal fistulae were defined as abnormal tubular connections between the anal canal/rectum and the skin. Fistula borders were delineated between T2 high signal and surrounding fibrosis or fat. All contents within the tract were segmented to calculate fistula volume using ITK‐Snap (Version 4.2.0). Scans were independently evaluated by two experienced gastrointestinal radiologists (PL and UP), blinded to both clinical data and chronological order of scans, who performed segmentation of MRI fistula volume and calculated the VAI.

### Reliability assessment

Inter‐rater reliability for MRI fistula volume measurements was assessed using intraclass correlation coefficients (ICC) derived from independent segmentations by two radiologists.

### Outcomes

The study addressed three key outcomes:

1. To provide additional evidence supporting the construct validity of CAF‐QoL by evaluating the association between MRI measures (fistula volume and VAI) of disease burden:
  - CAF‐QoL
  - CAF‐QoL domain subscales
  - Short‐Form CAF‐QoL
2. To evaluate the association between MRI measures (fistula volume and activity indices [VAI]) and established clinical activity indices (PDAI) and global QoL measures (Equation 5D).
3. To assess the reliability and reproducibility of MRI fistula volume as a measure of disease activity in pfCD.

### Statistical analysis

Statistical analyses were performed using IBM SPSS Statistics (Version 29; IBM Corp., Armonk, NY, USA). Continuous variables are presented as median (interquartile range [IQR]) unless otherwise specified. Associations between MRI fistula volume, MRI activity indices and PROMs were evaluated using Spearman's rank correlation coefficients (*ρ*). Between‐group comparisons (e.g. active vs. remission [6]) were performed using Mann–Whitney *U* tests. Receiver operating characteristic analyses evaluated discriminatory performance of MRI measures in identifying impaired QoL thresholds. Inter‐rater reliability for volumetric assessment was evaluated using intraclass correlation coefficients (ICC). All statistical tests were two‐tailed, with statistical significance defined as *p* < 0.05.

## RESULTS

### Study cohort

Thirty‐eight patients with pfCD were recruited, all of whom had a baseline MRI performed within 3 months (median 0.5 months, IQR 0–3) of completing a CAF‐QoL questionnaire, providing paired baseline MRI and CAF‐QoL measurements. Of these, 18 patients had both a follow‐up CAF‐QoL questionnaire and concurrent MRI performed within 3 months (median 1 month, IQR 1–2.5), providing paired baseline and follow‐up MRI and CAF‐QoL measurements. Baseline demographic and clinical characteristics are summarised in Table 2. Median age was 38 years (IQR 31–43) and 52.6% were female. Median Crohn's disease duration was 132 months (IQR 84–216), and median fistula duration was 84 months (IQR 60–120). Most patients were receiving advanced medical therapy at baseline (89.5%), and 31.6% were on concomitant immunomodulators. The cohort predominantly demonstrated complex perianal disease (86.8%), with transsphincteric tracts being the most common fistula subtype (57.9%) (Table S1). Abscess or cavity formation was observed in 21.1% of patients, and horseshoe or supralevator extension each occurred in 18.4%. Median MRI fistula volume at baseline was 859.5 mm^3^ (IQR 107–1968) (Table S2).

**TABLE 2:** Patient demographics.

Characteristic	Value
Age, years	38 (31–43)
Sex, *n* (%)	
Female	20 (52.6)
Male	18 (47.4)
Ethnicity, *n* (%)	
White (Northern European)	31 (81.6)
Asian (other)	6 (15.8)
Black African	1 (2.6)
Weight, kg	76.9 ± 14.6
Smoking status, *n* (%)	
Non‐smoker	28 (73.7)
Ex‐smoker	9 (23.7)
Current smoker	1 (2.6)
Crohn's disease duration, months	132 (84–216)
Fistula duration, months	84 (60–120)
On biologic therapy at baseline, *n* (%)	34 (89.5)
On immunomodulator at baseline, *n* (%)	12 (31.6)
Previous medication exposure, *n* (%)	
≥1 previous medication	22 (57.9)
≥2 previous medications	12 (31.6)
≥3 previous medications	6 (15.8)
≥4 previous medications	2 (5.3)
Previous surgeries, number	4 (3–6)
Previous fistula repairs, number	1 (0–2)
Previous luminal surgery, *n* (%)	9 (23.7)

### MRI disease activity across pfCD classes

Following classification of patients using TOpClass criteria [18], subclass‐specific median values for MRI metrics are presented in Table S2. Median average VAI scores increased stepwise across anatomical complexity. This gradient is consistent with increasing tract inflammation and branching in more severe disease and replicates findings from a previous study [11]. A marked rise in fistula volume was observed with class escalation, consistent with previous data [11]. Volumes increased more than ten‐fold between Class 1 and Class 2 subclasses, with the highest burden in Class 3 (although *n* = 1), reflecting anatomical complexity.

### Associations between MRI metrics and clinical/QoL measures

Average MRI fistula volume (mean of 2 readers' scores) demonstrated strong, statistically significant FDR‐adjusted correlations with CAF‐QoL Total (*ρ* = 0.40, *p* = 0.03), CAF‐QoL Domain A (*ρ* = 0.52, *p* = 0.004), CAF‐QoL Domain C (*ρ* = 0.35, *p* = 0.048) and PDAI (*ρ* = 0.59, *p* < 0.001) (Table 3). The VAI showed no significant associations with CAF‐QoL subscales or PDAI after FDR correction. This pattern is consistent with biological expectations: a larger fistula burden (measured by fistula volume) is associated with increased fistula‐related symptoms recorded by the CAF‐QoL questionnaire (CAF A: symptoms; CAF‐C: quality of life) and global perianal disease activity (PDAI). Spearman correlation analysis demonstrated no significant associations between MRI fistula volume and individual EQ‐5D domains, including mobility (*ρ* = 0.12, *p* = 0.49), self‐care (*ρ* = −0.14, *p* = 0.40), usual activities (*ρ* = 0.22, *p* = 0.18), pain/discomfort (*ρ* = 0.15, *p* = 0.39) or anxiety/depression (*ρ* = 0.30, *p* = 0.068). Similarly, MRI volume showed a weak, non‐significant correlation with Equation 5D Level Sum Score (*ρ* = 0.27, *p* = 0.11). A weak inverse trend was observed with EQ‐5D VAS (*ρ* = −0.32, *p* = 0.06), though this did not reach statistical significance.

**TABLE 3:** Associations between MRI fistula imaging metrics and clinical/quality‐of‐life outcomes (CAF‐QoL/Equation 5D/PDAI Total).

MRI volume metric	CAF‐A	CAF‐B	CAF‐C	CAF‐QoL Total	CAF short form	Equation 5D VAS	PDAI
Average Fistula Volume	0.52 (0.004)*	0.30 (0.07)	0.35 (0.048)*	0.40 (0.03)*	0.37 (0.04)*	−0.32 (0.06)	0.59 (0.0009)***
Average VAI Score	0.28 (0.33)	0.027 (0.87)	0.12 (0.65)	0.13 (0.65)	0.10 (0.65)	−0.22 (0.43)	0.33 (0.29)

*Note*: Spearman correlations (*ρ*) between MRI fistula volume metrics and clinical/ QoL outcomes with FDR‐adjusted *p*‐values in brackets. *p*‐values were adjusted using the Benjamini–Hochberg false discovery rate (FDR). Significant FDR‐corrected values (pFDR ≤0.05) are starred.

* Statistically significant.

*** Very highly statistically significant.

### MRI metrics and QoL remission

Following dichotomization of the cohort into ‘remission’ (defined by a CAF‐QoL score less than or equal to 37) or ‘active disease’ (defined by a CAF‐QoL score greater than 37), analysis was restricted to baseline observations (*n* = 38) to avoid repeated measures bias from multiple timepoints. Patients in CAF‐QoL remission had significantly lower MRI fistula volumes than those with active disease (279 vs. 965 mm^3^, *p* = 0.04; Cohen's *d* = 0.52), whereas VAI did not differ significantly between groups (13 vs. 13, *p* = 0.22; Cohen's *d* = 0.34) (Figure 1).

**FIGURE 1:**
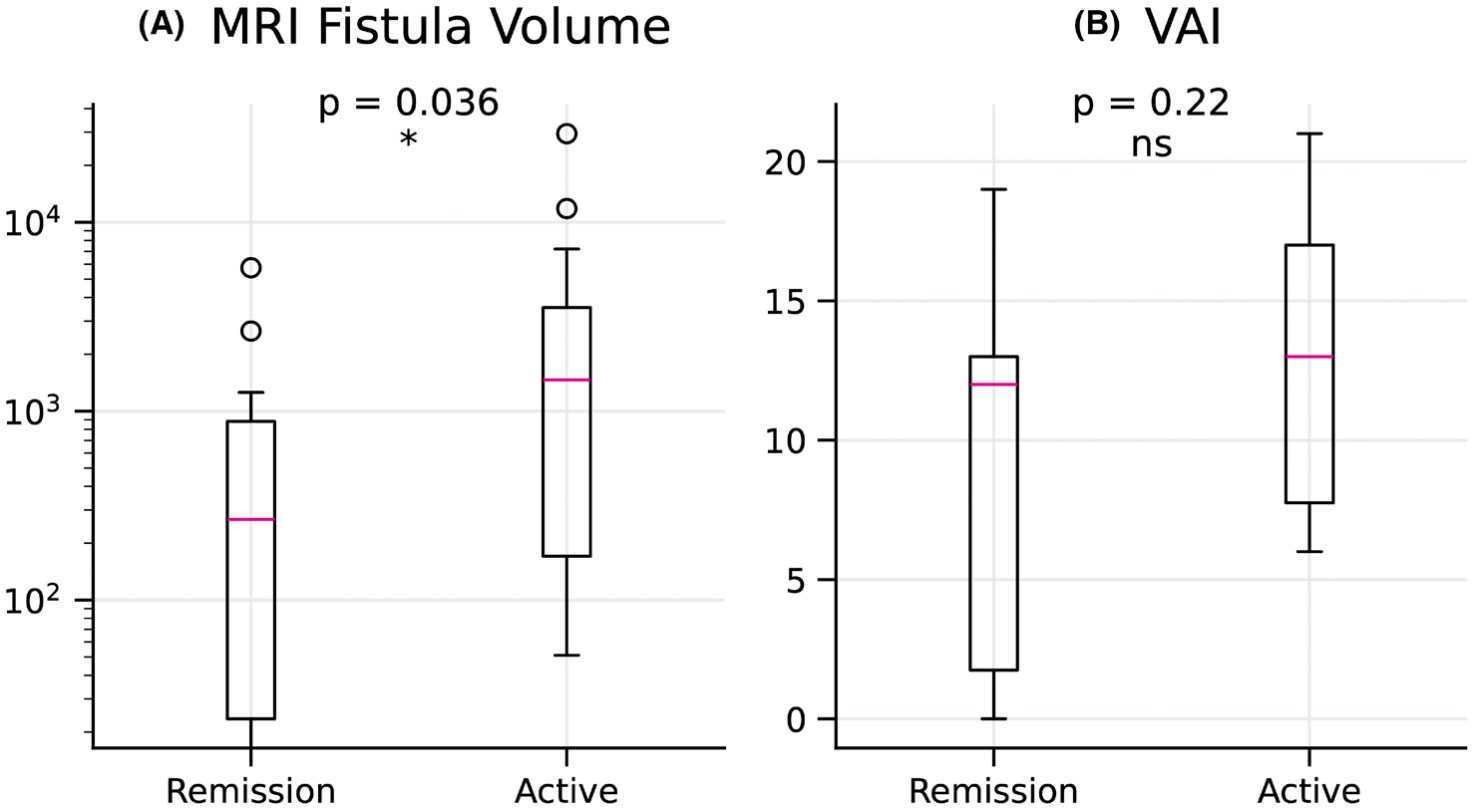
Boxplots showing average MRI fistula volume in remission vs. active disease and average VAI score in remission vs. active disease. Remission defined as CAF‐QoL Total ≤37 at the same timepoint. Values are median [IQR]. Primary test = Mann–Whitney *U* (two‐sided). MRI fistula volume: significantly higher in active disease, with moderate effect sizes (*d* = 0.52). VAI: higher in active vs. remission, but not statistically significant (small–moderate *d* = 0.34).

### Longitudinal associations

Paired data were available for 18/38 patients who completed both baseline and follow‐up CAF‐QoL with a contemporaneous MRI scan. In view of non‐parametric distributions and the ordinal structure of the VAI, Spearman's rank correlation was used as the primary method for assessing monotonic associations. Percentage changes in fistula volume from baseline strongly correlated with changes in PDAI (*ρ* = 0.74, *p* ≤ 0.01), outperforming VAI (Table 4). Percentage volume change correlated with change in CAF‐QoL Total (*r* = 0.61, *p* = 0.02). VAI change showed a moderate association with ΔCAF‐QoL Total (*ρ* = 0.50, *p* = 0.04). Neither volume change nor VAI change was significantly associated with a change in EQ 5D score.

**TABLE 4:** Longitudinal associations between change in MRI fistula volume and VAI change vs. clinical/QoL outcomes.

Outcome	Predictor	Spearman *ρ* (*p*)
CAF‐QoL Total	Δ fistula volume	0.17 (0.52)
	% MRI fistula volume change	0.34 (0.24)
	Δ VAI	0.50 (0.04*)
Short Form CAF‐QoL	Δ fistula volume	0.10 (0.70)
	% MRI fistula volume change	0.21 (0.47)
	Δ VAI	0.36 (0.15)
PDAI	Δ fistula volume	0.61 (0.01*)
	% MRI fistula volume change	0.74 (0.003**)
	Δ VAI	0.49 (0.047*)
EQ 5D‐VAS	Δ fistula volume	−0.31 (0.24)
	% MRI fistula volume change	−0.22 (0.47)
	Δ VAI	−0.29 (0.27)

*Note*: In addition to Spearman's rank correlations, Pearson correlation coefficients were also calculated as a sensitivity analysis to assess linear relationships. For CAF‐QoL outcomes, Pearson correlations for percentage change in MRI fistula volume were *r* = 0.61 (*p* = 0.02) for CAF‐QoL Total and *r* = 0.54 (*p* = 0.046) for Short Form CAF‐QoL. Pearson correlations for Δ VAI were *r* = 0.21 (*p* = 0.43) for CAF‐QoL Total and *r* = 0.20 (*p* = 0.44) for Short Form CAF‐QoL. Results for PDAI and EQ‐5D VAS were consistent with Spearman analyses and are not shown separately due to concordance in direction and magnitude.

* Statistically significant.

** Highly statistically significant.

### ROC analysis

ROC Curve analysis with Youden's cut‐off was used to identify the optimal MRI volume threshold for predicting quality of life remission vs. active disease, defined by a CAF‐QoL cutoff of ≤37 (remission). MRI fistula volume demonstrated modest discriminative performance for CAF‐QoL remission (AUC 0.67). Optimal thresholds (~1,255 mm^3^) prioritised sensitivity (0.88) over specificity for discriminating between active disease impairing quality of life and remission (Figure 2).

**FIGURE 2:**
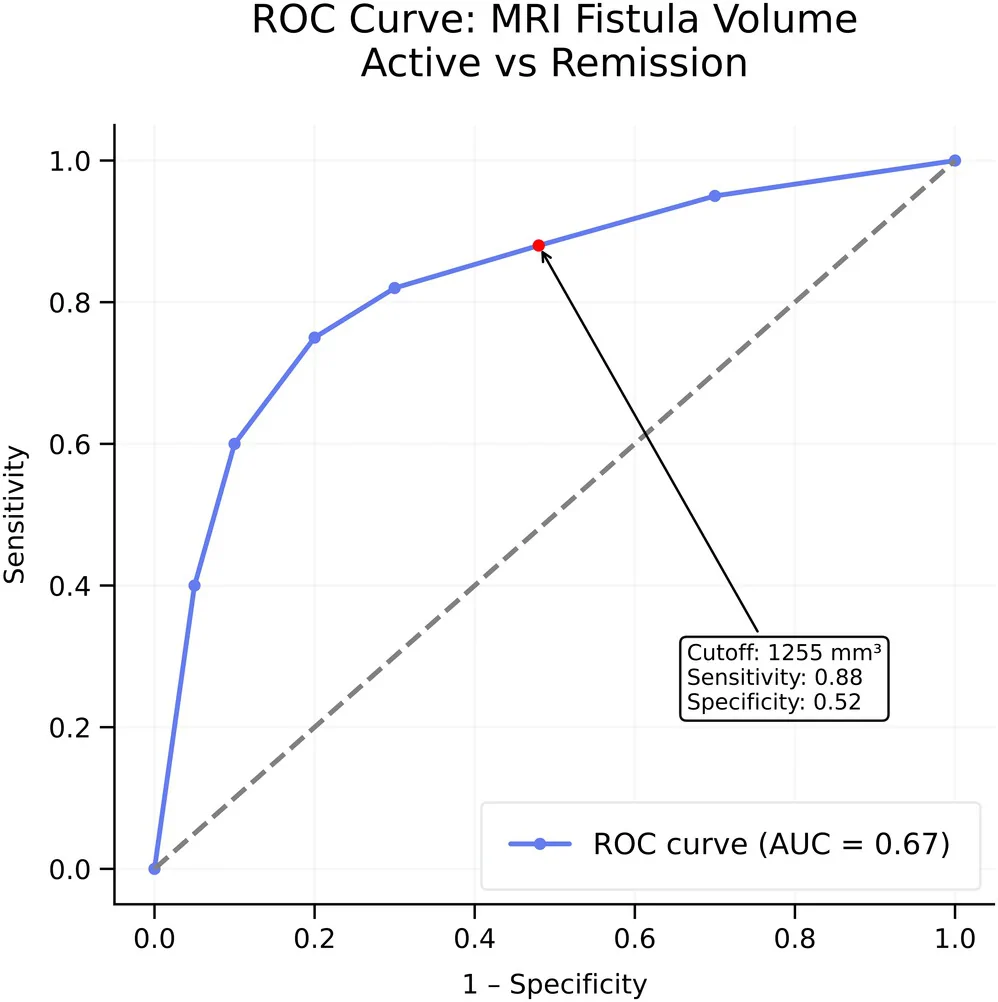
ROC curve showing optimal cut‐off value for discrimination of remission vs. active disease using MRI fistula volume. The optimal AUC was obtained from the larger group of baseline readings with a Youden Cutoff of 1255 mm^3^ identified as discriminating between remission and active disease (i.e. >1255 mm^3^ indicates active disease). AUC 95% CI from bootstrap (4,000 samples); sensitivity/specificity CIs also bootstrapped at the Youden cutoff.

### Reliability of MRI fistula volume

Inter‐observer agreement for continuous MRI fistula volume measurements was assessed using a two‐way absolute agreement intraclass correlation coefficient. Agreement between readers was excellent at both baseline (ICC 0.995) and follow‐up (ICC 0.989), demonstrating high reproducibility of volumetric assessment. Median time to measure fistula volume was 125 s (IQR 97–177 s).

## DISCUSSION

This prospective single‐centre study at a tertiary referral centre for complex IBD demonstrates that quantitative MRI fistula volume reflects both clinical disease activity and disease‐specific quality of life in pfCD, providing objective construct validation of the CAF‐QoL questionnaire. By linking an imaging biomarker with a validated patient‐reported outcome measure, our findings address two internationally recognised priorities in perianal Crohn's disease research: improving imaging assessment and better measuring the patient experience [15]. Whilst CAF‐QoL has previously undergone psychometric validation using IBDQ and MAGNIFI as comparators, showing limited correlation with the latter [6], objective imaging measures such as MRI fistula volume have not been systematically evaluated as comparators. This is one of the earliest studies to link radiological parameters directly with CAF‐QoL, thereby aligning imaging assessment with patient‐centred outcomes [5, 15].

Patients with pfCD frequently rely on MRI findings to understand their disease, and greater disease engagement is associated with improved outcomes [20, 21, 22]. Importantly, MRI fistula volume captured aspects of disease burden that were meaningful to patients. Baseline MRI fistula volume correlated strongly with clinical activity (PDAI; *ρ* = 0.59, *p* < 0.001) and moderately with CAF‐QoL (*ρ* = 0.395, *p* = 0.03), particularly Domain A (symptoms; *ρ* = 0.515, *p* < 0.01). Fistula volume may also distinguish active from remission states, unlike the VAI. The absence of significant associations between the VAI and either PDAI or CAF‐QoL is consistent with previous reports demonstrating limited responsiveness of semiquantitative MRI indices, although findings from this study should be interpreted with caution given the modest sample size [6]. These findings suggest that MRI‐derived fistula volume captures the clinical and patient‐reported burden of pfCD, supporting the need for further research validating its use with CAF‐QoL for disease assessment and the development of patient‐centred endpoints in future trials.

Accepting the limitations of a relatively small subset of patients with paired follow‐up questionnaires and MRI (*n* = 18), longitudinal changes in MRI‐derived fistula volume correlated strongly with changes in clinical disease activity (ΔPDAI; *ρ* = 0.74, *p* ≤ 0.01) and moderately with changes in disease‐specific quality of life (ΔCAF‐QoL; *ρ* = 0.61, *p* = 0.02), providing supportive evidence for the responsiveness and construct validity of CAF‐QoL. Whilst previous studies established its psychometric properties, our findings demonstrate convergence between longitudinal changes in a quantitative imaging biomarker and a disease‐specific patient‐reported outcome, supporting the responsiveness of both volumetric assessment and CAF‐QoL to evolving disease. ROC analysis demonstrated moderate discrimination for identifying CAF‐QoL remission, supporting the potential role of MRI volumetry as a monitoring biomarker (AUC 0.67); a threshold of 1,255 mm^3^ provided high sensitivity (88%) but modest specificity (52%). External validation of these thresholds is required to determine clinically meaningful cut‐off values.

Inter‐observer reliability for volumetric measurement was excellent (ICC 0.995 at baseline; 0.989 at follow‐up), supporting the reproducibility of the described workflow. The volumetric method used here is accessible through the open source software, ITK‐SNAP, and quick with average calculation times of around 2 min per scan. Inter‐observer agreement was excellent at both time points, indicating that fistula volume is a robust imaging biomarker when applied by trained radiologists. This suggests feasibility for broader adoption in both research and clinical workflows. Future multicentre work should focus on external validation, inter‐radiologist standardisation and integration of volumetrics into routine MRI reporting frameworks, alongside exploration of automated or semi‐automated segmentation approaches to improve scalability [23].

Our findings extend previous work showing that MRI fistula volume reflects anatomical disease severity and fistula drainage by demonstrating, for the first time, an association with disease‐specific quality of life [10, 24, 25]. Whilst radiological healing of fistulae in pfCD is accepted as a hard endpoint [26], and has been shown to predict long‐term sustained clinical remission [27, 28], the intermediate states between baseline disease and complete healing are less well defined. Prior studies have shown MRI volumetry to correlate with disease severity (TOpClass classification [11]; increasing fistula volume correlates with a worse disease class), with fistula drainage activity (improved/remission states) [13], and offer early signals that quantitative changes can anticipate clinical outcomes [29]. Within this context, our results demonstrate that volume also correlates with QoL (CAF‐QoL), bridging a crucial gap between radiological burden and patient‐centred outcomes which has previously only been described in PISA, when complete radiological healing was associated with improved symptom burden [28]. Moreover, subclass analyses in our cohort confirmed stepwise increases in both VAI and especially volume across anatomical classes, with volumes rising more than ten‐fold from Class 1 to Class 2, and peaking in Class 3, consistent with the expected increase in tract complexity, branching and inflammatory burden.

Whilst these results require validation in larger, prospective, multicentre studies, a single volume number that rises and falls with symptoms is an intuitive metric that may facilitate shared decision‐making and enhance patient engagement in the future [21, 22, 30]. Used together, volumetry and CAF‐QoL could help distinguish persistent inflammatory burden from symptomatic improvement alone, inform multidisciplinary treatment decisions and provide more meaningful endpoints for clinical trials. Pending further work to integrate volumetry into routine MRI reporting and the outcome of subsequent validation studies, clinicians may find volumetry helpful when evaluating treatment response, determining suitability for or success of down‐staging or ‘rationalisation’ procedures, assessing whether a biologic therapy is effective or confirming the impact of surgical intervention.

### Limitations

This study has several limitations. It was conducted at a single tertiary referral centre with a predominance of complex, treatment‐refractory disease (21% with abscesses/cavities and >80% with complex fistulae), which may limit generalisability. Although QoL assessments were obtained within a three‐month window around MRI, the median intervals were short (2 weeks at baseline and 4 weeks at follow‐up) and no patient had intervention in between completion of the questionnaire and their MRI scan; nevertheless, the partially‐contemporaneous timing may have attenuated associations given the inherently fluctuating nature of symptoms and the disease. Volumetry demonstrated excellent inter‐observer reliability, but all readers were experienced gastrointestinal radiologists, requiring validation in centres with varying expertise. The cohort was modest (*n* = 38) and incomplete follow‐up CAF‐QoL data reduced power for longitudinal analyses despite efforts to minimise attrition through patient reminders. Consequently, the absence of statistically significant associations, including between MRI activity indices and CAF‐QoL, should be interpreted cautiously and does not exclude a clinically meaningful relationship. Although both absolute and percentage changes in fistula volume were analysed, percentage change was selected as the primary dynamic marker of disease activity to remain consistent with previous studies of MRI fistula volume [11, 13]. We acknowledge that percentage change may be disproportionately influenced by small baseline volumes; however, in our cohort, patients with smaller‐volume disease generally demonstrated relatively small changes in volume, whereas those with more extensive, high‐volume disease tended to retain a greater disease burden during follow‐up. No significant association was found between radiological findings and the EQ‐5D, perhaps reflecting the more nuanced nature of CAF‐QoL regarding the specific impact of fistula‐related symptoms on QoL. Finally, volumetry was not compared directly with contrast‐enhanced indices such as MAGNIFI because routine contrast‐enhanced MRI is not standard practice at our centre or many UK institutions. Larger multicentre studies including a broader spectrum of disease severity are needed to confirm these findings.

### Future work

Future multicentre studies should validate MRI volumetry across different scanners, protocols, radiologists and healthcare systems. Automated segmentation using deep learning may further improve scalability and facilitate incorporation into routine MRI reporting and future therapeutic trials [23].

## CONCLUSION

This study provides objective construct validation of the CAF‐QoL questionnaire using quantitative MRI fistula volume. MRI volumetry demonstrated excellent reproducibility and reflected both clinical disease activity and patient‐reported disease burden. These findings support the complementary use of quantitative MRI biomarkers and disease‐specific patient‐reported outcomes for assessing pfCD and developing patient‐centred endpoints for future clinical trials. External validation in larger, multicentre cohorts is required.

## AUTHOR CONTRIBUTIONS

**Easan Anand:** Conceptualization; investigation; writing – original draft; methodology; validation; visualization; writing – review and editing; software; formal analysis; project administration; data curation. **Theo Pelly:** Conceptualization; investigation; methodology. **Uday Patel:** Conceptualization; investigation; methodology; validation; visualization; writing – review and editing; data curation; formal analysis. **Rory Callan:** Conceptualization; investigation; methodology; data curation. **Reshma Kanani:** Data curation; conceptualization; investigation. **Ailsa Hart:** Conceptualization; investigation; writing – review and editing; supervision. **Phil Tozer:** Conceptualization; investigation; writing – original draft; data curation; supervision. **Phillip Lung:** Conceptualization; investigation; writing – review and editing; validation; visualization; methodology; formal analysis; data curation; supervision.

## FUNDING INFORMATION

No specific funding was received for this study. All potential conflicts of interest were managed, and no funders influenced the study design.

## CONFLICT OF INTEREST STATEMENT

AH: has served as consultant, advisory board member or speaker for AbbVie, Arena, Atlantic, Bristol‐Myers Squibb, Celgene, Celltrion, Falk, Galapogos, Lilly, Janssen, MSD, Napp Pharmaceuticals, Pfizer, Pharmacosmos, Shire and Takeda. She also serves on the Global Steering Committee for Genentech. PT: Speaker fees and/or advisory board for Takeda, J&J, Tillots, Ferring and Falk. The remaining co‐authors have no conflicts of interest.

## ETHICS STATEMENT

Ethical approval for this study was granted with R&D reference RD21/071 IRAS 308386. Confirmation of capacity and capability was provided by London North West University Healthcare NHS Trust. Patients provided informed consent to enter the Fistula outcomes database study and were free to withdraw at any time.

## PATIENT AND PUBLIC INVOLVEMENT

No patients were directly involved in the development of this study.

## PROVENANCE AND PEER REVIEW

Not commissioned, externally peer reviewed.

## Supporting information


**Table S1.** Fistula Characteristics (Clinical and radiological findings).
**Table S2.** pfCD Classes 1, 2a, 2b, 3.

**Appendix S1.** STROBE Statement—Checklist of items that should be included in reports of observational studies.

## Data Availability

Anonymised data has been provided without compromising data protection and confidentiality throughout.
