# Supplementary material for: Objective validation of the Crohn's Anal Fistula Quality of Life questionnaire using quantitative MRI fistula volume in perianal fistulising Crohn's disease
Source: Colorectal Dis. 2026 Oct 17;28(10):e70653. doi: 10.1111/codi.70653 (PMC13675774; doi:10.1111/codi.70653)
Supplement: Supplementary file 1 [file CODI-28-0-s001.docx]

**Supplementary Tables**

***Supplementary Table 1. Fistula Characteristics (Clinical and radiological findings)***

| Characteristic | n (%) |
| --- | --- |
| *Montreal Age Classification (A)* |  |
| A1 | 3 (7.9) |
| A2 | 30 (78.9) |
| A3 | 5 (13.2) |
| *Montreal Location (L)* |  |
| L1 | 8 (21.1) |
| L2 | 16 (42.1) |
| L3 | 13 (34.2) |
| L1+L4 | 1 (2.6) |
| *Montreal Behaviour (B)* |  |
| B1 | 25 (65.8) |
| B2 | 9 (23.7) |
| B3 | 1 (2.6) |
| Missing | 3 (7.9) |
| *Parks Classification* |  |
| Intersphincteric (1) | 9 (23.7) |
| Transsphincteric (2) | 22 (57.9) |
| Suprasphincteric (3) | 1 (2.6) |
| Extrasphincteric (4) | 6 (15.8) |
| Horseshoe extension | 7 (18.4) |
| Supralevator extension | 7 (18.4) |
| New tract identified | 3 (7.9) |
| Associated cavity/abscess | 8 (21.1) |
| Simple fistula | 5 (13.2) |
| Complex fistula | 33 (86.8) |
| Number of external openings | Median 1 (1–2) |
| MRI Fistula Volume | Median 859.5 mm^3^ (IQR 107 – 1968) |

***Supplementary Table 2: pfCD Classes 1, 2a, 2b, 3***

| **PfCD Class** | **Number of patients** | **VAI Score** | **Fistula Volume, mm³** |
| --- | --- | --- | --- |
| 1 | 15 | 8.5 [0.0 – 12.0] | 117.0 [0.0 – 264.0] |
| 2a | 7 | 11.5 [10.5 – 14.0] | 1,165.5 [463.5 – 2,749.0] |
| 2b | 15 | 15.5 [11.5 – 17.25] | 1,369.0 [997.75 – 4,966.0] |
| 3 | 1 | 16.5 [16.5 – 16.5] | 12,715.0 [12,715.0 – 12,715.0] |
|  |  |  |  |

*Scores and fistula volume are presented as median values with IQR in brackets.*
