# Supplementary material for: Objective validation of the Crohn's Anal Fistula Quality of Life questionnaire using quantitative MRI fistula volume in perianal fistulising Crohn's disease
Source: Colorectal Dis. 2026 Oct 17;28(10):e70653. doi: 10.1111/codi.70653 (PMC13675774; doi:10.1111/codi.70653)
Supplement: Supplementary file 2 [file CODI-28-0-s002.docx]

**STROBE Statement—Checklist of items that should be included in reports of observational studies**

**Manuscript Title:** *Evaluating the Association Between MRI Fistula Volume and Patient-Reported Quality of Life in Perianal Fistulising Crohn's Disease: A Prospective Single-Centre Study*

| **Item** | **Recommendation** | **Page(s) in Manuscript** |
| --- | --- | --- |
| **1a** | Indicate the study's design with a commonly used term in the title or the abstract | Title; Abstract |
| **1b** | Provide in the abstract an informative and balanced summary of what was done and what was found | Abstract |
| **2** | Explain the scientific background and rationale for the investigation | Introduction |
| **3** | State specific objectives, including any prespecified hypotheses | Introduction (Study Aim/Objectives) |
| **4** | Present key elements of study design early in the paper | Methods – Study Design |
| **5** | Describe the setting, locations, and relevant dates, including periods of recruitment, exposure, follow-up, and data collection | Methods – Study Setting and Participants |
| **6a** | Give the eligibility criteria and the sources and methods of selection of participants. Describe methods of follow-up | Methods – Participants |
| **6b** | For matched studies, give matching criteria | Not applicable |
| **7** | Clearly define all outcomes, exposures, predictors, potential confounders and effect modifiers | Methods – Outcomes; MRI Evaluation; Clinical Assessment |
| **8** | Give sources of data and details of methods of assessment for each variable | Methods – MRI Acquisition; MRI Evaluation; PROMs; PDAI |
| **9** | Describe any efforts to address potential sources of bias | Methods; Discussion – Limitations |
| **10** | Explain how the study size was arrived at | Methods – Study Design/Statistical Analysis |
| **11** | Explain how quantitative variables were handled in the analyses | Statistical Analysis |
| **12a** | Describe all statistical methods, including those used to control for confounding | Statistical Analysis |
| **12b** | Describe methods used to examine subgroups and interactions | Statistical Analysis; Results (CAF-QoL remission and subgroup analyses) |
| **12c** | Explain how missing data were addressed | Statistical Analysis |
| **12d** | Explain how loss to follow-up was addressed | Statistical Analysis; Discussion – Limitations |
| **12e** | Describe any sensitivity analyses | Statistical Analysis; Results (Pearson sensitivity analysis) |
| **13a** | Report numbers of individuals at each stage of the study | Results – Study Cohort; Longitudinal Cohort |
| **13b** | Give reasons for non-participation at each stage | Results; Discussion – Limitations |
| **13c** | Consider use of a flow diagram | Participant flow diagram provided (or Not applicable if omitted) |
| **14a** | Give characteristics of study participants and information on exposures and potential confounders | Results – Table 1 |
| **14b** | Indicate number of participants with missing data for each variable of interest | Results; Statistical Analysis |
| **14c** | Summarise follow-up time | Methods; Results |
| **15** | Report numbers of outcome events or summary measures over time | Results; Tables 2–4 |
| **16a** | Give unadjusted estimates and, where applicable, adjusted estimates and their precision | Results; Tables 2–4 |
| **16b** | Report category boundaries when continuous variables were categorised | Methods (CAF-QoL remission defined as <37); Results |
| **16c** | Translate estimates of relative risk into absolute risk, if relevant | Not applicable |
| **17** | Report other analyses done, including subgroup, interaction and sensitivity analyses | Results (ROC analysis, reliability analysis, subgroup analyses, sensitivity analyses) |
| **18** | Summarise key results with reference to study objectives | Discussion |
| **19** | Discuss limitations, taking into account potential sources of bias or imprecision | Discussion – Limitations |
| **20** | Give a cautious overall interpretation considering objectives, limitations, multiplicity of analyses and other evidence | Discussion; Conclusion |
| **21** | Discuss the generalisability (external validity) of the study results | Discussion – Limitations; Future Work |
| **22** | Give the source of funding and the role of the funders | Funding Statement |

**Notes**

- This study is reported in accordance with the STROBE Statement for observational cohort studies.
- Items marked "Not applicable" were not relevant to the design of this prospective observational cohort study.
